# Serum proteomic analysis of novel predictive serum proteins for neurological prognosis following cardiac arrest

**DOI:** 10.1111/jcmm.16201

**Published:** 2020-12-18

**Authors:** Shuang‐shuang Gu, Jin Li, Min Jiang, Yi Zhou, Bing Yang, Kehui Xie, Yun‐fei Jiang, Xin‐rui Jiang, Fei He, Jun Wang

**Affiliations:** ^1^ Department of Emergency Nanjing Drum Tower Hospital The Affiliated Hospital of Nanjing University Medical School, Nanjing Jiangsu China; ^2^ Nanjing Jiangbei New Area Biopharmaceutical Public Service Platform Co. Ltd, Nanjing Jiangsu China; ^3^ Department of Emergency Nanjing Drum Tower Hospital Clinical College of Nanjing Medical University, Nanjing Jiangsu China

**Keywords:** cardiac arrest, LC‐MS/MS, neurological outcome, proteomics, serum protein biomarkers

## Abstract

Early prognostication of neurological outcome in comatose patients after cardiac arrest (CA) is vital for clinicians when assessing the survival time of sufferers and formulating appropriate treatment strategies to avoid the withdrawal of life‐sustaining treatment (WLST) from patients. However, there is still a lack of sensitive and specific serum biomarkers for early and accurate identification of these patients. Using an isobaric tag for relative and absolute quantitation (iTRAQ)‐based proteomic approach, we discovered 55 differentially expressed proteins, with 39 up‐regulated secreted serum proteins and 16 down‐regulated secreted serum proteins between three comatose CA survivors with good versus poor neurological recovery. Then, four proteins were selected and were validated via an enzyme‐linked immunosorbent assay (ELISA) approach in a larger‐scale sample containing 32 good neurological outcome patients and 46 poor neurological outcome patients, and it was confirmed that serum angiotensinogen (AGT) and alpha‐1‐antitrypsin (SERPINA1) were associated with neurological function and prognosis in CA survivors. A prognostic risk score was developed and calculated using a linear and logistic regression model based on a combination of AGT, SERPINA1 and neuron‐specific enolase (NSE) with an area under the curve of 0.865 (*P* < .001), and the prognostic risk score was positively correlated with the CPC value (R = 0.708, *P* < .001). We propose that the results of the risk score assessment not only reveal changes in biomarkers during neurological recovery but also assist in enhancing current therapeutic strategies for comatose CA survivors.

## 1 INTRODUCTION

To date, more than one million out‐of‐hospital cardiac arrest (OHCA) sufferers are newly diagnosed each year worldwide, which leads to a substantial public health burden as a result of its high morbidity and mortality.^1^ Despite the great progress achieved in resuscitation and critical care management, the prognosis of OHCA sufferers remains poor. Brain injury, including hypoxic‐ischaemic brain injury (HIBI) and ischaemia‐reperfusion brain injury, is a major cause of death among comatose OHCA patients after the restoration of spontaneous circulation (ROSC).^2^, ^3^ Survivors of cardiac arrest (CA) often require lengthy intensive care admission, rehabilitation and ongoing therapy of chronic complications because of poor functional outcome.^4^, ^5^ Thus, early and accurate identification of neurological outcome among CA patients is vital for clinicians when assessing the survival time of sufferers and formulating appropriate treatment strategies to avoid the withdrawal of life‐sustaining treatment (WLST) from patients who may have a good neurological outcome and the use of futile treatments in patients who may have a poor neurological outcome.

Repeated clinical examination in combination with electroencephalography, electrophysiological tests, computed tomography (CT) and magnetic resonance imaging (MRI) are generally employed for early judgments of neurological outcome.^6^ However, the operation of most of these devices requires great skill and expertise for accurate judgment,^7^ and the evidence derived from these methods is not consistent.^8^, ^9^ In addition, neuron‐specific enolase (NSE), a dimeric isoform of the glycolytic enzyme enolase found mainly in neurons, is present in serum following different brain disorders.^10^ NSE levels have been found to increase in comatose CA survivors with severe brain injury, and the levels of NSE at 72 h after CA have been regarded as an effective prognostic marker, according to the European Resuscitation Council and European Society of Intensive Care Medicine (ERC‐ESICM) guidelines for prognostication after CA.^11^, ^12^ Targeted temperature management (TTM) in combination with several drugs has become the standard treatment for comatose CA patients, as TTM effectively improves the recovery of neurological function; nevertheless, the above‐mentioned data originated from a cohort study performed prior to the widespread implementation of TTM. False‐positive rates have been reported for nearly one‐third of comatose CA patients after TTM.^13^ Furthermore, newly developed biomarkers such as protein S‐100B do not add any real value to current prognostication models with NSE.^14^ Therefore, the development of neuronal injury biomarkers must be approached from a new angle so that clinical therapeutic strategies for comatose CA survivors can be improved.

Recent successes reported in the literature have illustrated the role of mass spectrometry‐based proteomics as an indispensable analytical tool in the workflow for disease biomarker discovery based on liquid biopsy.^15^ In our work, we performed an investigation of serum proteins that were differentially expressed in comatose CA survivors with good versus poor neurological recovery using an isobaric tag for relative and absolute quantitation (iTRAQ)‐based proteomic approach. Differentially expressed proteins were validated via an enzyme‐linked immunosorbent assay (ELISA) approach in a larger‐scale independent sample, and a prognostic risk score was developed and calculated using a linear and logistic regression model based on a combination of select proteins and NSE. We propose that the results of the risk score assessment not only reveal changes in biomarkers during neurological recovery but also assist in enhancing current therapeutic strategies for comatose CA survivors.

## 2 MATERIALS AND METHODS

### 2.1 Patient sample collection

From January 2014 to December 2019, we conducted a retrospective observational study of OHCA patients at Nanjing Drum Tower Hospital (Nanjing, China). The participants were biologically unrelated, and informed written consent was obtained before sample collection. The exclusion criteria were as follows: failure to achieve the ROSC; age < 18 years; past history of irreparable brain damage; the recovery of consciousness after the ROSC without undergoing TTM; death within 72 h after the ROSC; did not receive TTM; and known haematological disease. Blood samples were immediately collected from venous blood puncture into EDTA tubes during the course of routine intensive care at 72 h after the ROSC. From each sample, 2 mL of blood was allowed to clot at 4°C for at least 2 h and then was centrifuged at 1000 × g for 30 min to precipitate blood cells. Then, the serum was collected and divided into aliquots followed by freezing at –80°C until analysis.

### 2.2 Proteomic digestion and iTRAQ labelling

Before the proteomic approach, a Human 14 Multiple Affinity Removal LC Column (Agilent, USA) was used to deplete highly abundant proteins according to the manufacturer's instructions. Then, concentrated serum samples were mixed with 1 × SDT lysis buffer (4% SDS, 100 mM Tris‐HCl, 1 mM DTT, pH 7.6) and then heated in boiling water for 15 min. After centrifugation at 14,000 rpm for 20 min, the supernatant was collected. Depleted serum protein concentrations were detected using a BCA protein assay kit (Pierce, USA). A total of 100 μg of sample was reduced and alkylated by a final concentration of 10 mM dithiothreitol and 50 mM iodoacetamide, respectively. Then, the digested samples with 2.5 μg of trypsin (Sigma, USA) were individually labelled with iTRAQ reagents at room temperature for 1 h. Finally, the six labelled peptide aliquots were combined for subsequent fractionation.

### 2.3 Strong cation exchange (SCX)

Tryptic peptides (100 μg) were loaded onto a polysulfoethyl column (4.6 × 100 mm, 5 μm, 200 Å) (PolyLC Inc, Maryland, USA) with an AKTA Purifier 100 (GE Healthcare, USA) and eluted using ACN/potassium phosphate KH_2_PO_4_ buffers (buffer A: 25% ACN/10 mM potassium phosphate, pH 3.0; buffer B: 25% ACN/80% 10 mM potassium phosphate, 500 mM potassium chloride, pH 3.0). The elution programme was 100% buffer A for 10 min, continued by a gradient of 0‐8% of buffer B for 22 min, followed by a gradient of 8‐52% for 25 min and a 52%‐100% gradient for 3 min. At the end, the gradient was maintained at 100% buffer B for 8 min and in buffer A for 10 min. Finally, 15 fractions of equal volume were collected, vacuum dried and stored at −80°C until liquid chromatography with tandem mass spectrometry (LC‐MS/MS) analysis.

### 2.4 LC‐MS/MS analysis and data analysis

A Q‐Exactive mass spectrometer (Thermo Scientific, USA) was used for LC‐MS/MS analysis. Formic acid (0.1%) and acetonitrile (4%, TEDIA, USA) buffer (4%) were mixed and added for dissolution before loading. An Acclaim PepMap100 (100 μm × 2 cm, 100 Å, 3 μm particle size) (Thermo Scientific, USA) following an EASY column (an Acclaim C18 PepMap100 nano‐Trap column (10 cm, ID75 μm, 3 μm particle size) (Thermo Scientific, USA)) was used to chromatographically separate the peptide mixture. Mass spectrometry analysis was performed in full‐scan mode (300‐1800 m/z) by an Orbitrap mass analyzer at a mass resolution of 70,000 at 200 m/z in the Q‐Exactive mass spectrometer. All tandem mass spectra were generated by the higher‐energy collision dissociation (HCD) approach. The analysis of acquired MS/MS spectra was performed for protein identification and quantification using Proteome Discoverer software (version 1.4) (Thermo Scientific, USA). Protein identification was carried out using the sequest algorithms to search the RefSeq human protein sequence database from the National Center for Biotechnology Information (NCBI) with the search parameters defined as follows: 10 ppm of mass tolerance or precursor ions and 0.1 Da for fragment ions. Trypsin was specified as the digesting enzyme, while two missed cleavages were permitted. Cysteine carbamidomethylation and modifications (N terminus and lysine residues) were defined as fixed modifications, and methionine oxidation was a variable modification. The results were filtered using the specifications of a q‐value of 0.01 and the use of only high‐confidence peptides with a false‐positive detection rate (FDR) <1% based on the target‐decoy approach. Quantification was performed using abundances of reporter ions based on signal‐to‐noise ratio values or intensity, while ratios were calculated using the average of unique peptides from proteins.

### 2.5 ELISA validation and measurement

Serum angiotensinogen (AGT), alpha‐1‐antitrypsin (SERPINA1), transgelin‐2 (TAGLN2) and Talin‐1 (TLN1) concentrations were determined by specific human ELISA kits (Abcam, USA), and experimental steps were performed according to the manufacturer's instructions. In addition, ELISAs were performed in a clinical laboratory to measure the NSE level.

### 2.6 Diagnostic scores for neurological outcome development

The serum level of a candidate protein detected by an ELISA was subjected to receiver operating characteristic (ROC) analysis to evaluate the sensitivity and specificity to distinguish a poor neurological outcome from a good neurological outcome and to elucidate the prognosis of neurological outcome in comatose CA survivors. Linear and logistic regression patterns were established for the risk score of neurological outcome diagnosis.

### 2.7 Statistical analysis

Statistical analysis was performed for the normal distribution and homogeneity of variance test with SPSS software 22.0. Student's t test (two tails) and a one‐way analysis of variance (ANOVA) were employed to examine the difference between subjects with a normal distribution and a homogeneity of the variance, while the Benjamini‐Hochberg approach was utilized to test the FDR. The Chi‐Square‐test or the Fisher exact test was used to compare categorical data. ROC curves were generated to differentiate a poor neurological outcome from a good neurological outcome by the standard method of sensitivity and specificity. The results are shown as the means ± the standard deviation (SD). The generally accepted level of significance was *P* < .05. The relative intensity value was acquired by comparing the intensity of a sample to that of the sample with the lowest intensity.

## 3 RESULTS

### 3.1 Patient characteristics

**FIGURE 1:**
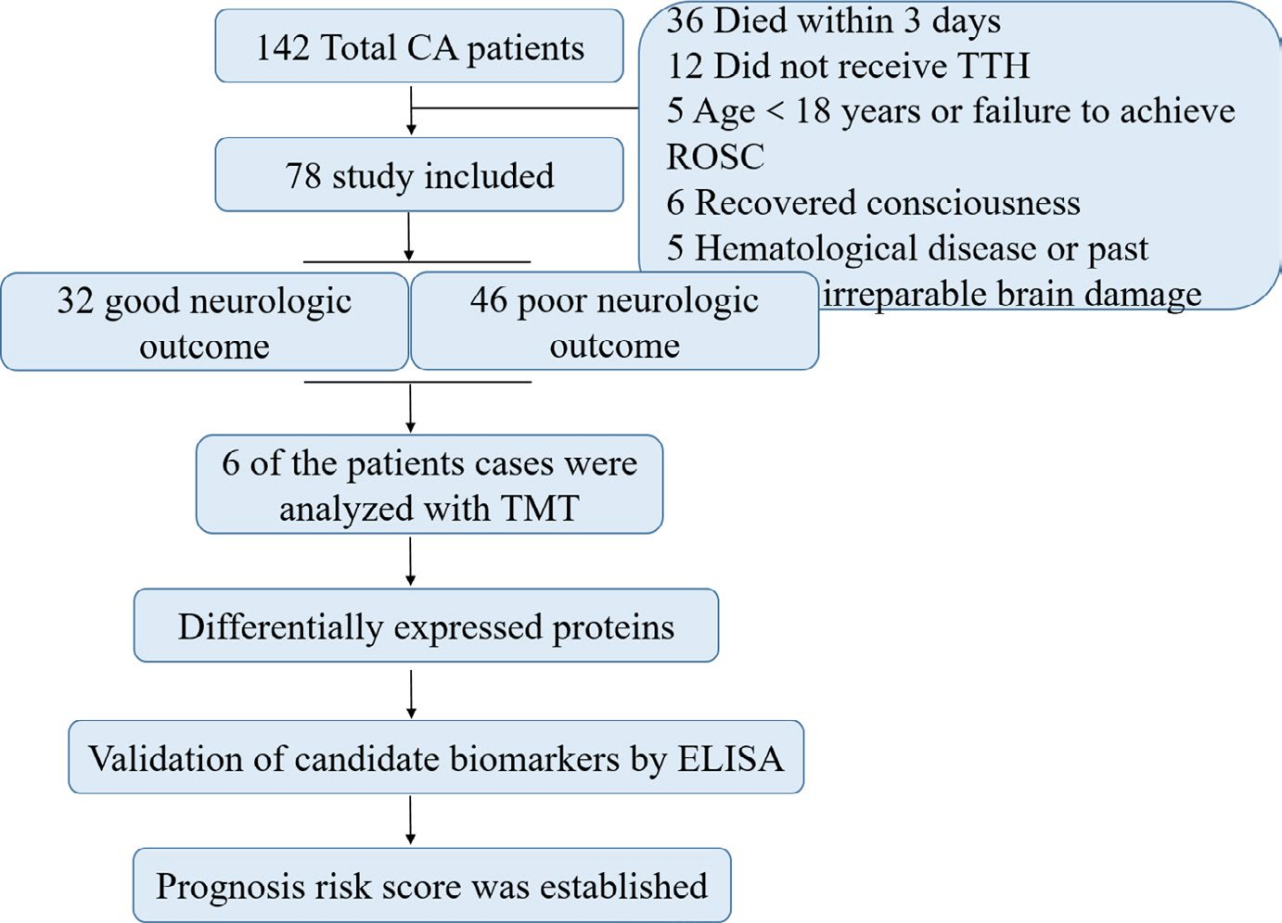
Outline of this workflow design

In the present work, we explored whether serum fingerprint proteins in early‐stage CA patients could predict neurological outcome. The early CA sufferers group included 78 participants who remained comatose 72 h after CA. Figure 1presents an outline of this workflow design. At 6 months post‐resuscitation, 32 patients had a Cerebral Performance Category (CPC) of 1‐2, 33 patients had a CPC of 3‐4, and 13 patients died during intensive care unit (ICU) stay or follow‐up (CPC of 5). All sufferers were divided into a good neurological outcome (GNO) group, defined as a CPC of 1‐2 (n = 32), and a poor neurological outcome (PNO) group, defined as a CPC of 3‐5 (n = 46). Then, three GNO patients and three PNO patients were randomly selected and assigned to these two groups mentioned above. Detailed demographics of the enrolled patients are presented in Table 1.

**TABLE 1:** Detailed demographics of the enrolled patients. Values are presented as median (interquartile range) or number (%).

Detailed demographics of the enrolled patients			
	GNO (CPC 1‐2) n = 32	PNO (CPC 3‐5) n = 46	*p* value
Age, years	52 (21‐70)	59 (33‐76)	0.008
Male, n (%)	25 (78.1)	30 (65.2)	0.218
Comorbidities, n (%)			
Hypertension	13 (40.6)	24 (52.1)	0.315
Diabetes	7 (21.8)	17 (36.9)	0.155
Hyperlipidaemia	5 (15.6)	10 (21.7)	0.5
Known IHD	5 (15.6)	12 (26.0)	0.27
Previous cerebral stroke	2 (6.2)	4 (8.6)	0.69
Witnessed arrest, n (%)	29 (90.6)	38 (82.6)	0.317
Bystander CPR, n (%)	27 (84.3)	36 (78.2)	0.5
First monitored rhythm, n (VF / VT) / (Asystole / PEA)	27 / 5	28 / 18	0.025
Time from OHCA to ROSC (min)	21 (11‐35)	35 (20‐57)	0.001
Cause of OHCA, n Cardiac/Non‐cardiac	29/3	42/4	0.91
GCS score	3 (3‐4)	3 (3‐3)	0.003
Initial serum lactate (mmol/L)	4.8 (2.3‐18)	6.5 (2.5‐23)	0.02

OHCA, out of hospital cardiac arrest; IHD, ischaemic heart disease; CPC, cerebral performance categories; CPR, cardiopulmonary resuscitation; VF, ventricular fibrillation; VT, ventricular tachycardia; PEA, pulseless electrical activity; ROSC, return of spontaneous circulation; GCS, Glasgow coma scale

### 3.2 Proteomic discovery using the iTRAQ approach

**FIGURE 2:**
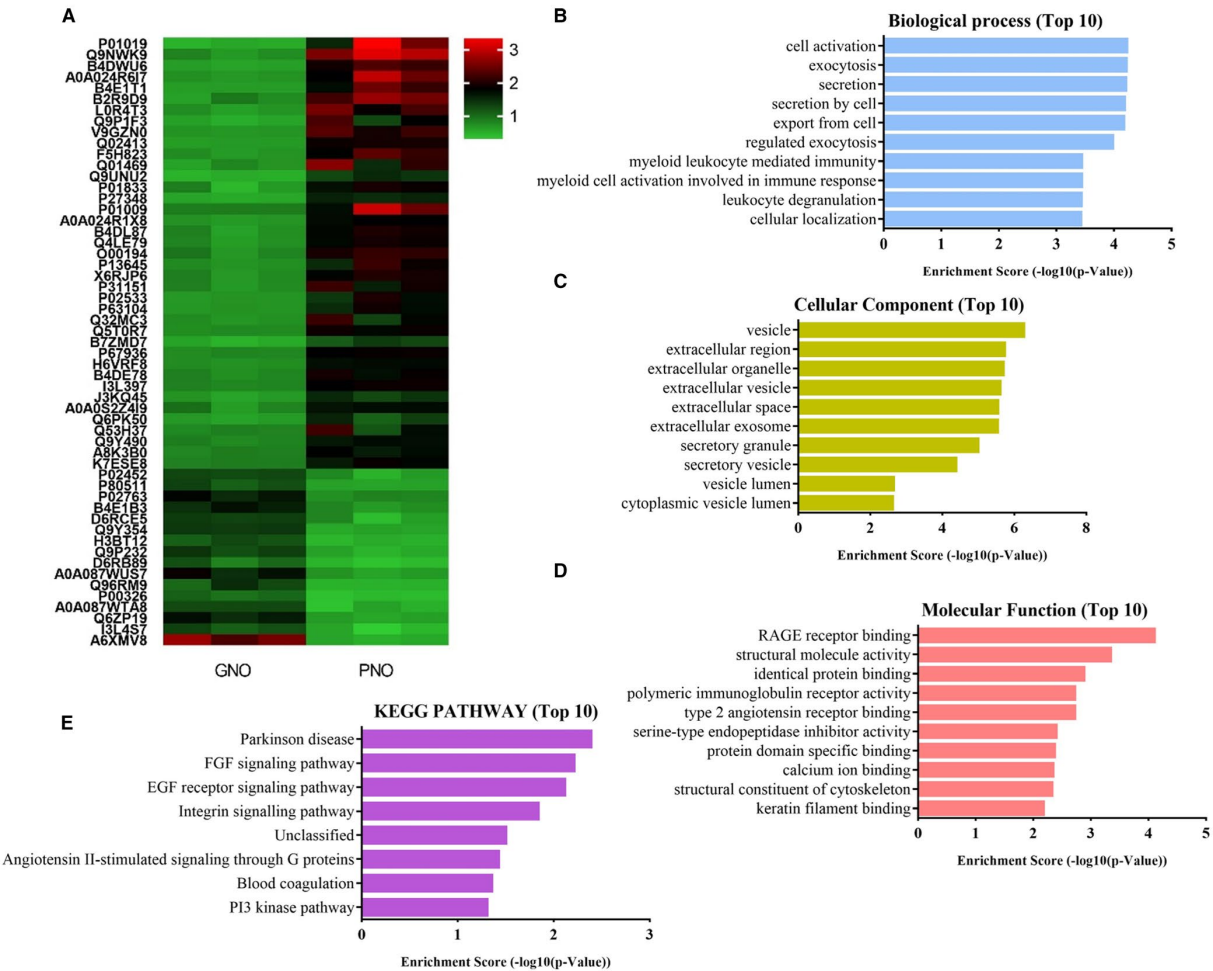
Differential protein expression characteristics and the exploration of potential functions of the dysregulation proteins. (A) up‐regulated and down‐regulated proteins (fold change ≥ 1.5) are coloured based on the heat map scale (up‐regulated: red, down‐regulated: green). The top ten GO analysis terms in the (B) biological process, (C) cellular component and (D) molecular function categories of dysregulation proteins between good neurological outcome and poor neurological outcome subjects. (E) Differentially expressed proteins were mapped to canonical pathways using the Kyoto Encyclopedia of Genes and Genomes tool

Subsequently, we carried out iTRAQ labelling‐based proteomics to identify differentially expressed proteins that are related to the prognosis of neurological recovery using serum samples collected from three GNO and three PNO patients. First, we depleted high‐abundance serum proteins by an immunodepletion kit before proteomic analysis because thousands of dynamic proteins from extremely high abundance to low abundance exist in serum. In total, 5,738 peptides that originated from 885 proteins were identified in the serum samples CA patients by LC‐MS/MS. By analysing the length of peptides and the molecular weight (MW) of proteins, we observed that the length of most peptide fragments was distributed in the range of 7‐15 amino acids, and more than 80% of the proteins were less than 100 kD (Data not shown). Among them, 55 proteins showed remarkable differences between the three GNO and three PNO patients (fold change ≥ 2 and *P* < .05), with 39 up‐regulated secreted serum proteins and 16 down‐regulated secreted serum proteins (Figure 2A). Then, the biological functions of the differentially expressed proteins (fold change ≥ 2 and *P* < .05) were surveyed using gene ontology and pathway investigation, including the biological process, cellular component and molecular function categories. From our data analysis, the top 10 biological processes (Figure 2B), cellular components (Figure 2C), molecular functions (Figure 2D) and pathways corresponding to differentially secreted serum proteins (Figure 2E) are presented.

### 3.3 Biomarker validation by ELISA and the predictive power of serum proteins for neurological prognosis

**FIGURE 3:**
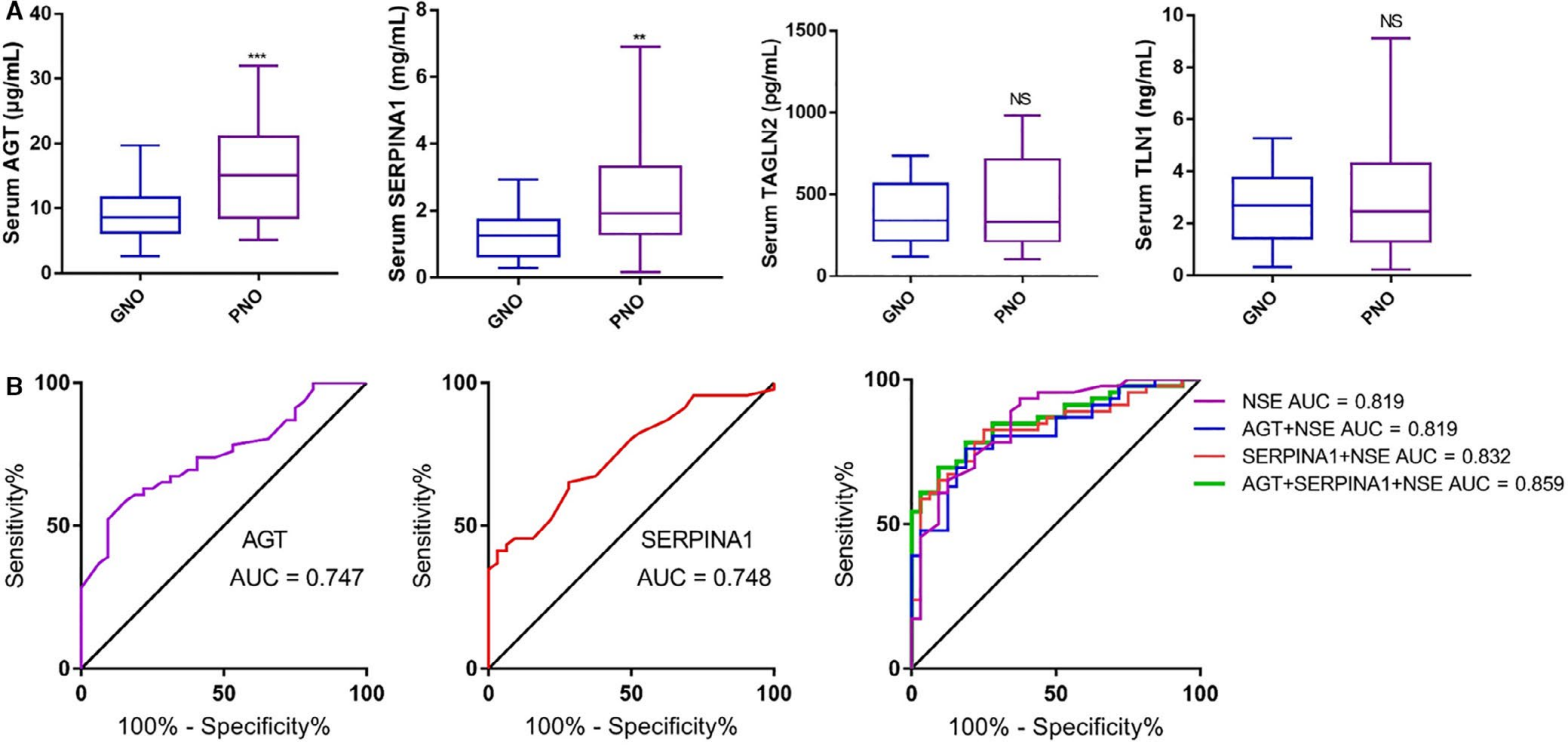
Validation of neurological outcome‐associated serum proteins. (A) Four up‐regulated proteins AGT, SERPINA1, TAGLN2 and TLN1 were detected using ELISA. (B) ROC curves were created to evaluate the power of dysregulated proteins (AGT, SERPINA1 and NSE) for predicting neurological outcome. Student's t test statistical analysis was used (NS, *P* > .05; **, *P* < .01 and ***, *P* < .001)

The validation of candidate protein biomarkers is crucial for clinical applications. A set of significantly differentially expressed proteins (AGT, SERPINA1, TAGLN2 and TLN1) in the cohorts (n = 78, 32 GNO patients and 46 PNO patients) were selected and measured using ELISAs. Selection was dependent on the following basic selection experimental guidelines: containing more than one unique peptide being identified and quantified, differential expression fold change ≥ 2, multiple hypothesis testing (FDR < 0.05) and significance based on a standard Student's t test (*P* < .05). As shown in Figure 3A, the serum concentrations of AGT and SERPINA1 were predominantly up‐regulated, whereas no significant difference in TAGLN2 or TLN1 was found in PNO patients. To evaluate the power of these differentially expressed serum proteins for the prediction of neurological prognosis, we performed a ROC curve analysis. The ROC curve for the results for AGT (Figure 3B) shows an area under the curve (AUC) of 0.747 (95% CI = 0.64 to 0.854), the ROC curve of the results for SERPINA1 show an AUC of 0.748 (95% CI = 0.641 to 0.854), the ROC curve of the results for TAGLN2 show an AUC of 0.548 (95% CI = 0.42 to 0.678) and the ROC curve of the results for TLN1 show an AUC of 0.52 (95% CI = 0.392 to 0.649). Overall, the results showed that AGT and SERPINA1 have the potential to serve as neurological prognosis biomarkers. To evaluate the power of NSE to predict neurological outcome, serum NSE was determined, and the ROC curve of the results for NSE showed an AUC of 0.819 (95% CI = 0.724 to 0.914) (Figure 3B). It has been widely assumed that the corresponding multiple effective biomarkers could be combined to generate a single score to assist clinicians in making a better diagnostic assessment,^16^ with an increase in the utilization of multiplex biomarkers. Thus, multivariate logistic regression analysis was carried out for these two markers combined with NSE. As shown in Figure 3B, the AUC of the combined results for NSE and AGT was 0.819 (95% CI = 0.728 to 0.911), and the AUC of the combined results for NSE and SERPINA1 was 0.832 (95% CI = 0.741 to 0.922), which was superior to that of the NSE biomarker alone. The AUC of the combined results of AGT, SERPINA1 and NSE was 0.859 (95% CI = 0.778 to 0.939); this combination showed the best prognostic efficiency with the highest sensitivity and specificity to differentiate a poor neurological prognosis from a good neurological prognosis.

### 3.4 Development of a prognostic panel for neurological outcome

**FIGURE 4:**
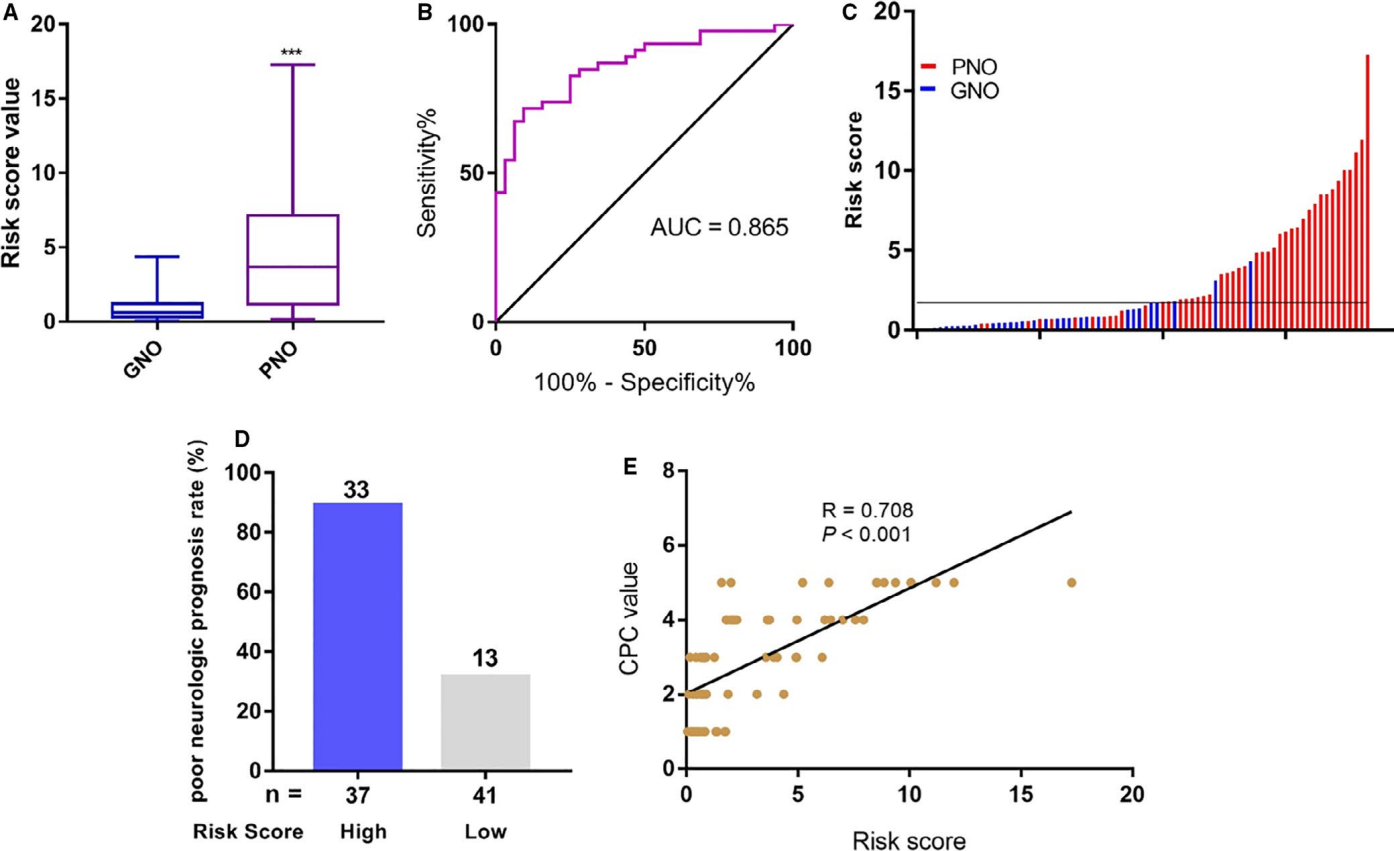
Distribution of the risk score and neurological outcome status in study. (A) Statistical analysis for distribution of risk score between good neurological outcome and poor neurological outcome patients with Student's t test (***, *P* < .001). (B) Waterfall plots for distribution of risk score and neurological outcome status of individual patients. (C) ROC curves were created to evaluate the power of risk score. (D) Poor neurological outcome rate between the high‐ and low‐score groups. (E) Correlation analysis between risk score and CPC value

To further elucidate the signature of the combination of AGT, SERPINA1 and NSE for predicting neurological prognosis, we constructed and calculated the characteristic risk score for each participant. According to the natural log of the intensity level of AGT, SERPINA1 and NSE, the following equation was generated and calculated for each individual using a linear and logistic regression model: ln(Risk score) = 1.26441 × ln(AGT)+1.30947 × ln(NSE)+ 0.69694 × ln(SERPINA1) −7.30787. Statistical analysis showed that the risk scores of the PNO group were significantly higher than those of the GNO group (Figure 4A). The sensitivity and specificity of the risk score for neurological outcome were 71.7% and 90.6%, respectively, with an AUC of 0.865 (95% CI = 0.769 to 0.932) (Figure 4B), while the threshold value (cut‐off point) was defined as 1.748. Subsequently, all CA participants were divided into low‐ and high‐risk score groups according to the threshold point value (1.748). The proportion of PNO individuals in the high‐risk score group was predominantly higher than that in the low‐risk score group according to an analysis of the association between the risk score distribution and the neurological outcome status (Figure 4C). Interestingly, patients with higher risk scores were in the PNO group. Further investigation showed that the high‐risk score group had a significantly higher rate of poor neurological outcome (89.19%, 33/37) than the low‐risk score group (31.70%, 13/41) (Figure 4D). Then, a linear correlation analysis demonstrated that the risk score was positively correlated with the CPC value (R = 0.708, *P* < .001) (Figure 4E).

## 4 DISCUSSION

Disturbances in neurocognitive performance are a core feature among CA survivors. Over the last few years, the therapeutic management of patients who are comatose after CA has been widely improved. However, few therapeutic interventions are available to completely alleviate neurological damage after CA[7]. Determining the prognosis of neurological outcome for comatose sufferers after CA is critical for personalized therapy and to assist in end‐of‐life medical decision‐making. On the other hand, the identification and evaluation of disease progression based on liquid biopsy are particularly encouraging sources of novel, easily available protein biomarkers as serum proteomics can reveal systemic alterations that occur with biological dysfunction.^17^ In the current study, we applied an iTRAQ labelling‐based approach coupled with LC‐MS/MS‐based proteomics to identify alterations in serum biomarkers among patients with different levels of neurological recovery. We discovered and confirmed that increased levels of AGT and SERPINA1 coupled with NSE compose a signature that is strongly associated with the prognosis of neurological outcome status. The results of this study emphasize the possible application of serum fingerprint proteins in predicting the neurological outcome of CA patients in the clinic.

The principal advantage of the use of this specific proteomic method to search for candidate biomarkers is that it permits the simultaneous measurement of multiple proteins within a single sample.^18^ In addition, it is not essential to define ahead of time which biomarkers should be analysed. For example, NSE, which is released into the bloodstream once the nervous system is injured, serves as a prognostic biomarker after CA. Recently, other potential biomarkers, including S100,^14^ B‐type natriuretic peptide,^19^ non‐coding RNA^20^ and cell‐free DNA,^21^ have been investigated. However, their suboptimal sensitivity and specificity limit their application.^22^ Moreover, the unifying principal rule of these prior research studies is that these proteins were known in advance. In several recent studies, increasing attention has been focused on the identification and description of disease progress by non‐invasive surrogate markers from fluid biopsy, such as serum or plasma. The advantages of fluid biopsy biomarkers include the following: a) the biomarkers can be detected in a non‐invasive way; b) fluid biopsy can be performed in ambulatory settings; and c) fluid biopsy is a reliable source of biomarkers and can be repeatedly analysed.^17^ Using serum proteomics as a "discovery‐based approach" to protein profiling in disease biomarker research has also been recommended recently.^23^ Indeed, previous work revealed novel predictive biomarkers that could be used to determine neurological recovery using two‐dimensional gel electrophoresis (2D‐GE) coupled with matrix‐assisted laser desorption/ionization‐time of flight (MALDI‐TOF) MS.^22^ In the present study, we compared the variation in serum protein differences using iTRAQ labelling‐based quantitative proteomics, and we identified additional proteins and more differentially expressed proteins than were reported in prior work.^22^ In this regard, the global‐tagging iTRAQ technique was more sensitive than 2D‐GE, as the comigration and partial comigration of proteins could compromise the repeatability and accuracy of quantification in the 2D‐GE approach.^24^

AGT, expressed as a constitutive protein by the liver and various other tissues, including the brain, interacts with renin to generate the prohormones angiotensin Ⅱ and angiotensin.^25^ An increasing amount of evidence has suggested that most AGT mRNA is expressed in the brain, comprising approximately one‐third of mRNAs originating in the liver that are present in ependymal and astrocytes.^26^, ^27^ A series of studies suggests that AGT produced by astrocytes could be constitutively secreted into perivascular space. However, the relative proportion of AGT secreted into the circulation versus that located intracellularly and the role of the secreted extracellular AGT are unknown. In addition, the existence of a high level of AGT in astrocytes may also indicate a more comprehensive function for the brain renin‐angiotensin system,^27^ and AGT is required for the initiation of the brain renin‐angiotensin system cascade, providing an impetus for a better understanding of its regulation in the brain.^28^ The relationship between the renin‐angiotensin system and brain function has been investigated and confirmed in previous work.^29^ SERPINA1, a member of a large family of molecules involved in complement activation, blood clotting and inflammatory responses, is also highly produced by the liver and secreted into the circulation. SERPINA1 deficiency results in a genetic disorder that damages the liver and lung as a result of uncontrolled neutrophil elastase and non‐functional SERPINA1 in the liver.^30^ In the past decade, an increasing amount of evidence has suggested that in addition to its broad‐spectrum antiproteinase activity, SERPINA1 has tissue‐protective properties and multiple anti‐inflammatory properties. For instance, SERPINA1 has been found to interfere with the activity of caspases 1 and 3, protecting pancreatic beta cells,^31^ lung alveolar and endothelial cells^32^, ^33^ and skin fibroblasts^34^ from the effects of apoptosis. In addition, SERPINA1 has been shown to down‐regulate the levels of nitric oxide, leukotriene B4 and pro‐inflammatory cytokines,^35^ including interleukin‐1b, interleukin‐6, interleukin‐8, interleukin‐32, tumor necrosis factor (TNF)‐a and monocyte chemoattractant protein‐1, without preventing the delivery of the anti‐inflammatory cytokine interleukin‐10 into the blood. SERPINA1 was also found to protect host cells from the influence of microorganisms, preventing their replication and infectivity.^36^ However, the fact that oxidants,^37^ metalloproteinases^38^ and the excision of its reactive site loop inactivate SERPINA1 by oxidation may cause it to lose some of its effectiveness under inflammatory conditions. Recently, SERPINA1 was reported to be a chaperone in amyloidoses, and it plays a role in amyloid aggregation, which may lead to the pathogenesis of amyloidotic diseases, for example amyotrophic lateral sclerosis (ALS), familial amyloid polyneuropathy (ATTR) and Alzheimer's disease.^39^, ^40^, ^41^ Previous findings have revealed that AGT belongs to another serine‐protease inhibitor (SERPIN) family member, SERPIN clade A, member 8. Although structurally resembling a SERPIN, the AGT of most species, except for that of lamprey, does not have the same function as the general inhibitory SERPIN.

This study has a few limitations, one of which is its small sample size of only 78 participants, including 32 GNO patients and 46 PNO patients with serum available for proteomic analysis, which is unfavourable for tests of the robustness of the model. Thus, the specificity and sensitivity of the diagnostic model should be evaluated with a larger number of patients and in a prospective study. In addition, a series of low‐abundance proteins, such as NSE, was not determined in our serum samples because the sensitivity of LC‐MS/MS‐based serum proteomics is remarkably influenced by high‐abundance proteins, leading to difficulties in the determination of low‐abundance proteins.

In brief, using iTRAQ labelling coupled with LC‐MS/MS‐based serum proteomics, we successfully identified a serum protein profile indicative of neurological recovery‐related proteins among comatose CA survivors. This profile can be used for the effective monitoring of these patients during the neurological prognosis phase. Based on these biomarkers, we experimentally tested and validated four differentially expressed proteins in several clinical samples using an ELISA method. On the basis of two selected differentially expressed proteins, AGT and SERPINA1, combined with NSE, a linear and logistic regression model was created, and a risk score was acquired to prominently differentiate poor neurological outcome from good neurological outcome. Our work demonstrated that this risk score based on the combination of selected serum biomarkers and NSE could contribute to early and accurate neurological outcome prognostication.

## CONFLICT OF INTEREST

The authors confirm that there are no conflicts of interest.

## AUTHORS’ CONTRIBUTION

F. H and J. W: Experiment conception and design; S. S. G, J. L, M. J and Y. Z: Experiments; Y. F. J and X. R. J: Data analysis; S. S. G: Manuscript writing.

## Data Availability

The data that support the findings of this study are available from the corresponding author upon reasonable request.
